# Protein arginine methyltransferase 6 mediates cardiac hypertrophy by differential regulation of histone H3 arginine methylation

**DOI:** 10.1016/j.heliyon.2020.e03864

**Published:** 2020-05-12

**Authors:** Vineesh Vimala Raveendran, Kamar Al-Haffar, Muhammed Kunhi, Karim Belhaj, Walid Al-Habeeb, Jehad Al-Buraiki, Atli Eyjolsson, Coralie Poizat

**Affiliations:** a Cardiovascular Research Program, King Faisal Specialist Hospital and Research Center, Riyadh, Saudi Arabia; b College of Medicine, Al Faisal University, PO Box 50927, Riyadh 11211, Saudi Arabia; c King Saud University, Riyadh, Saudi Arabia; d Heart Centre, King Faisal Specialist Hospital and Research Center, Riyadh, Saudi Arabia; e Masonic Medical Research Institute, Utica, NY 13501, USA

**Keywords:** Proteins, Biochemistry, Molecular biology, Pathophysiology, Cardiology, PRMT6, Protein arginine methyltransferase 6, Histone H3 arginine methylation, Cardiac hypertrophy, Phenylephrine, Pressure overload hypertrophy, Heart failure, Epigenetics

## Abstract

Heart failure remains a major cause of hospitalization and death worldwide. Heart failure can be caused by abnormalities in the epigenome resulting from dysregulation of histone-modifying enzymes. While chromatin enzymes catalyzing lysine acetylation and methylation of histones have been the topic of many investigations, the role of arginine methyltransferases has been overlooked. In an effort to understand regulatory mechanisms implicated in cardiac hypertrophy and heart failure, we assessed the expression of protein arginine methyltransferases (PRMTs) in the left ventricle of failing human hearts and control hearts. Our results show a significant up-regulation of protein arginine methyltransferase 6 (PRMT6) in failing human hearts compared to control hearts, which also occurs in the early phase of cardiac hypertrophy in mouse hearts subjected to pressure overload hypertrophy induced by trans-aortic constriction (TAC), and in neonatal rat ventricular myocytes (NRVM) stimulated with the hypertrophic agonist phenylephrine (PE). These changes are associated with a significant increase in arginine 2 asymmetric methylation of histone H3 (H3R2Me2a) and reduced lysine 4 tri-methylation of H3 (H3K4Me3) observed both in NRVM and *in vivo*. Importantly, forced expression of PRMT6 in NRVM enhances the expression of the hypertrophic marker, atrial natriuretic peptide (ANP). Conversely, specific silencing of PRMT6 reduces ANP protein expression and cell size, indicating that PRMT6 is critical for the PE-mediated hypertrophic response. Silencing of PRMT6 reduces H3R2Me2a, a mark normally associated with transcriptional repression. Furthermore, evaluation of cardiac contractility and global ion channel activity in live NRVM shows a striking reduction of spontaneous beating rates and prolongation of extra-cellular field potentials in cells expressing low-level PRMT6. Altogether, our results indicate that PRMT6 is a critical regulator of cardiac hypertrophy, implicating H3R2Me2a as an important histone modification. This study identifies PRMT6 as a new epigenetic regulator and suggests a new point of control in chromatin to inhibit pathological cardiac remodeling.

## 1 Introduction

Heart failure (HF) is the most common reason for hospitalization and death worldwide in older adults [1]. HF is often preceded by ventricular hypertrophy, characterized by an increase in cell size associated with ventricular remodeling and increased fibrosis. Traditionally, cardiac hypertrophy is viewed as an initial compensatory mechanism to normalize wall stress [2, 3, 4, 5]. However, persistent exposure to stress as occurs in hypertension leads to cardiac wall dilatation and heart failure [3]. During the transition from adaptive to maladaptive hypertrophy, and finally to heart failure, many cellular pathways become activated resulting in profound changes in gene expression driven by specific transcription factors and remodeling of chromatin [6, 7, 8, 9, 10, 11, 12]. Chromatin conformation is regulated by histone modifiers that modify the histone “tails” and histone globular domain post-translationally to achieve proper packing of the genome within the cell nucleus. Recent studies indicate that abnormal modification of histone H3 (e.g. methylation or acetylation) can cause aberrant gene expression resulting in major cardiac pathologies including heart failure [13, 14, 15, 16, 17, 18]. Histone methyltransferases are dysregulated in the left ventricle of patients with dilated cardiomyopathy [16] and in heart failure [19, 20] suggesting a role of these enzymes in chromatin organization in humans. Likewise, excessive *de novo* mutations in genes regulating histone methylation contribute to severe forms of congenital heart defects [21]. Studies performed in mice have revealed that dysregulation of histone methyltransferases or demethylases results in major cardiac diseases *in vivo*. For example, a reduction in the expression of the histone demethylase Jumonji Domain-Containing Protein 2A (JMJD2A) protects against cardiac hypertrophy induced by TAC in mice, while transgenic mice over-expressing JMJD2A have increased cardiac hypertrophy [22]. Mono/trimethylation of histone H3 at lysine 4 and trimethylation of H3 at lysine 27 regulate the expression of genes controlling cardiomyocyte differentiation [20]. Overall, these studies demonstrate the role of lysine methyltransferases in cardiac hypertrophy and heart development. However, little is known on the role of protein arginine methyltransferases (PRMTs) in cardiac diseases.

PRMTs are a class of enzymes that add methyl groups to the guanidino nitrogen atoms of arginine residues in target proteins. To date, eleven PRMTs have been identified in the mammalian genome, classified into three subclasses based on the type of methylation they catalyze-symmetric or asymmetric dimethylation or monomethylation of arginine [23, 24]. Type I PRMTs catalyze asymmetric di-methylation, while type II mediates symmetric methylation of the nitrogen atom of arginine. Type III PRMT such as PRMT7, is responsible for mono-methylation of arginine residues. PRMTs control important cellular functions [25, 26]. Over expression of PRMTs is observed in many types of cancers [24]. Besides one recent report documenting the role of PRMT1 in heart failure [27], the function of PRMT members in heart disease remains unknown. PRMT6 specifically methylates arginine 2 of histone H3 (H3R2Me2a) and arginine 3 of histones H4/H2A [28, 29, 30]. H3R2Me2a causes demethylation of lysine 4 of histone H3 (H3K4me3), which inhibits the recruitment of mixed lineage leukemia (MLL)/WDR5 complex at the Hox2A gene and antagonizes H3K4Me3-mediated gene activation in breast cancer cells [29]. In the present study, we addressed the role of PRMT6 in cardiac hypertrophy. We show that PRMT6 protein expression is elevated in patients with end-stage heart failure and in mice subjected to TAC-induced cardiac hypertrophy. Phenylephrine (PE) treatment increases PRMT6 protein expression in isolated primary neonatal rat ventricular myocytes (NRVM), which correlates with enhanced H3R2Me2a and reduced H3K4Me3. Specific silencing of PRMT6 inhibits the pro-hypertrophic effect of PE on atrial natriuretic peptide (ANP) expression as well as on cell size. Importantly, knockdown of PRMT6 inhibits the spontaneous contraction rate of NRVM as well as the length and intervals of extracellular field potentials, indicating that PRMT6 is critical for cardiomyocyte hypertrophy and for cardiac contractility. These data suggest that modification of the epigenome secondary to changes in PRMT6 expression underlies pathological cardiac remodeling.

## 2 Materials and methods

### 2.1 Collection of human heart tissue

All procedures were performed under an approved protocol (RAC# 2100 024) from the Office of Research Affairs at King Faisal Specialist Hospital & Research Centre, Riyadh, Saudi Arabia. Patients with end-stage heart failure secondary to idiopathic dilated cardiomyopathy followed at the Heart Centre were enrolled in our study under informed consent. Hemodynamic function was monitored in all patients with dilated cardiomyopathy to monitor the progression of the cardiomyopathy and just prior to the cardiac transplantation. Immediately after the surgery, explanted hearts were frozen in liquid nitrogen and transferred to the laboratory with the help of the transplant coordinator. Control human hearts were obtained from donors who had died from accidental death or from incidents other than cardiac diseases and that could not be transplanted because of incompatibility with the recipient. Human hearts were stored at -80 °C until further molecular analysis. When ready for analysis, human hearts were thawed on ice and the same anatomical regions of the left ventricles were dissected. Functional data were unfortunately not available for control donor hearts.

### 2.2 Animals and transverse aortic constriction (TAC)

C57BL/6 mice were bred at the Comparative Medicine Department at King Faisal Specialist Hospital and Research Centre under a protocol approved by the Institutional Animal Care and Use Committee. For TAC experiments, 8–10 weeks old male mice were used. The aorta was ligated between the brachiocephalic artery and the left common carotid artery using a 7–0 polypropylene suture (Ethicon) to induce stenosis and pressure on the left ventricle. Sham surgery was performed on control animals by opening and closing the chest without aortic ligation. After 10, 21 and 42 days, mice were euthanized and hearts were collected and immediately frozen in liquid nitrogen for further biochemical analysis.

### 2.3 Echocardiography in mice

Cardiac dimension and function were measured in all animals at 10, 21- and 42-days post-Sham or TAC surgery using a VividE9 high-resolution imaging system (GE). Mice were subjected to 1–2% isoflurane for sedation before assessing cardiac function. Left ventricular dimensions were measured along a parasternal long-axis view and recorded in M-mode. End diastolic diameter (EDD) and end systolic diameter (ESD) were measured in order to calculate fractional shortening (FS) and ejection fraction (EF) and to determine left ventricular volume during diastole and systole.

### 2.4 Antibodies and plasmid DNA

Anti-PRMT6 antibody was purchased from Cell signaling technologies (cat# 14641S, Beverly, USA) and Abcam (Cat# ab104834, ab190902, Cambridge, USA). ANP (cat# SC-515701) and GAPDH antibodies were from Santa Cruz (cat#, sc-25778, California, USA). GATA-4 was purchased from Cohesion bioscience (cat# CPA1462, London, UK). H3R2Me2a (cat# MBS2520233) and H3K4Me3 (cat# MBS9385277) were from MyBioSource (San Diego, CA, USA). Phalloidin conjugated to TRITC was purchased from Sigma Aldrich (cat# FAK100). Total histone H3 antibody was purchased from Abcam (cat# ab201456, Cambridge, USA). PRMT6 expression vector was purchased from Origene (Prmt6 (NM_001106466), rat tagged ORF Clone).

### 2.5 Primary neonatal rat ventricular myocytes isolation and treatment

Primary neonatal rat ventricular myocytes (NRVM) were isolated using the Worthington isolation kit [32]. Briefly, 2–3 days old pups were sacrificed by decapitation and after opening the thorax, hearts were quickly removed. Cardiomyocytes were isolated by digestion with 0.5 mg/ml Type II collagenase and 0.5% Trypsin. After dissociation, the cells were pre-plated for 45–60 min to allow fibroblasts to attach and then plated on 100-mm cell culture dishes at a density of 3 × 10^6^ cells or onto 6-well dishes at 1 × 10^6^ in DMEM with high glucose (Invitrogen) supplemented with 10% fetal calf serum and antibiotics (100 U/ml penicillin, 10 mg/ml streptomycin solution). Cells were maintained in a humidified CO_2_ (5%) chamber at 37 °C for all experiments.

### 2.6 PRMT6 silencing and overexpression experiments

NRVM plated at a density of 1 × 10^6^ cells/well in 6-well plates were transfected with stealth siRNA (Cat# LOC100911617-RSS341517, RSS341518 and RSS341519, Invitrogen) using RNAimax transfection reagent according to the manufacturer's protocol. NRVM were left in serum-free media or treated with 100μM of hypertrophic agonist phenylephrine (PE) for 48 h. Total cell lysates were prepared for Western blot analysis. For PRMT6 over-expression, NRVM were transfected with PRMT6 expression vector using Lipofectamine 3000 according to the manufacturer's protocol. 48 hours post-transfection, cells were either processed for indirect immunofluorescence staining or for Western blot analysis.

### 2.7 Protein extraction

Approximately 50–100 mg of LV human tissue or mouse whole hearts were grinded in liquid nitrogen and protein were extracted in a high salt buffer (1M Hepes, 5M NaCl, 0.5M EDTA, and 2M sucrose) with a protease and phosphatase inhibitor cocktail (Thermo Scientific, Philadelphia, PA, USA). After lysis, tissue or cell extracts were sonicated to solubilize proteins. Soluble fractions were recovered by centrifugation at 12,500 rpm at 4 °C for 20 min.

### 2.8 Western blotting

Protein lysate concentrations were measured by Bradford assay (Bio-Rad). Aliquots of the supernatants normalized for protein concentrations were mixed with equal volumes of 2x SDS sample buffer and boiled at 100 °C for 5 min. Forty micrograms of heart or cell extract were analyzed on 4–12 % NuPAGE® Novex® Bis-Tris Gels (Invitrogen, USA). After transfer of the proteins onto PVDF membranes, membranes were blocked in TBS-T (50 mM Tris, 138 mM NaCl, 2.7 mM KCl, 0.05% Tween 20, pH 8.0) containing 5% nonfat dry milk at room temperature for 1 h. After washing, membranes were incubated with the indicated primary antibodies (1:500–1000 dilution) in TBS-T overnight at 4 °C. After washing three times in TBS-T membranes were incubated for 1 h with a corresponding secondary antibody in the dark at room temperature. Signals were detected using chemiluminescence and quantitated using ImageQuant LAS 4000 mini analyzer (GE Healthcare). After stripping of the membranes, Western blot was performed with GAPDH antibody for normalization and Adobe Photoshop CS6 for digitization.

### 2.9 Immunofluorescence and phalloidin staining

After the indicated PE treatment, NRVM were fixed in formalin and permeabilized with ice-cold methanol. Anti-α-actinin (1:800) or anti-PRMT6 (1:100) antibodies were incubated for 1 h at room temperature followed by incubation with secondary antibodies conjugated to tetramethylrhodamine (TRITC) or fluorescein isothiocyanate (FITC) for 1 h in the dark. After washing with PBS, Vectashield antifade mounting medium with DAPI was used for counterstaining the nucleus. Fluorescent images were visualized and analyzed using a Zeiss axioimager Z2 fluorescent microscope. Phalloidin staining was used to measure NRVM size in PRMT6 silenced cells. NRVM were fixed in 4% formaldehyde in PBS and then were incubated with TRITC conjugated phalloidin probe for F-actin. After washing, images were visualized and analyzed using a Nikon Ti100 fluorescent microscope. NRVM sizes were measured using Adobe Photoshop CS6 measurement tool.

### 2.10 Quantification of cardiomyocyte contractility and electrophysiological characteristics

The RTCA CardioECR system (ACEA Bioscience Inc. San Diego, CA) was used to study the effect of PRMT6 silencing on NRVM beating rate and electrophysiological characteristics. This system uses microelectrodes to record impedance readout to measure the spontaneous beating activity of cardiac cells [31]. CardioECR plates were coated with fibronectin at 37 °C for 3 h. NRVM were plated at a density of 30,000 viable cells per well. 24 hours after plating the cells, PRMT6 siRNA or Control siRNA was transfected after which NRVM were treated with 100 μM PE for 48 h. The impedance data were recorded every 10 min along with global field potential to measure beating rates and extracellular field potentials. Data were analyzed using the RTCA Cardio software to calculate spontaneous beating rates, amplitude and beating periods [31].

### 2.11 Statistical analysis

Statistical analysis was performed using Graphpad Prism 6 software. For echocardiographic data analysis, the Holm-Sidak method was used for multiple t-tests between the Sham and TAC groups at different time points, without assuming a consistent standard deviation (SD). Multiple unpaired t-Test, and one-way ANOVA or two-way ANOVA followed by Dunnet's or Tukey's multiple comparison post-hoc tests were used wherever applicable. Graphical data are represented as the mean ± SD. *p* values less than 0.05 or 0.01 were considered statistically significant.

## 3 Results

### 3.1 PRMT6 is increased in the LV of dilated human hearts and after pressure-overload hypertrophy in mice

To identify chromatin remodeling enzymes differentially regulated in failing human hearts, we collected LV tissue samples of patients in end-stage heart failure who underwent heart transplantation. Control donor hearts unsuitable for transplant were used as controls. Echocardiographic data was unfortunately not available for the control donor hearts. Echocardiographic data of the patients who underwent heart transplant showed a severely compromised cardiac function as indicated by the ejection fractions (EF) (average = 15.5 ± 4.1%) which was significantly below normal ranges (EF = 55–70%) (Table 1). Left ventricular end-diastolic diameters (LVEDD) were indicative of dilatation in patients with heart failure with averages of 6.78 ± 0.65 cm.

**Table 1:** Echocardiographic parameters of patients with heart failure prior to the heart transplant. Cardiac echocardiography was performed in all patients with heart failure just prior to the heart transplantation. Echocardiographic data was not available for control subjects. Ejection fraction, left ventricular end diastolic pressure (LVEDD), right ventricular end diastolic pressure (RVEDD) are shown. RVEDD was not available for patient 4.

Echocardiographic measurements	Patients				
	1	2	3	4	5
Ejection fraction (%)	9	13.1	9.1	31.6	15
LVEDD (cm)	6.7	7.5	7.7	4.3	7.7
RVEDD (cm)	3.8	3.5	4	N/A	4.1

**Figure 1:**
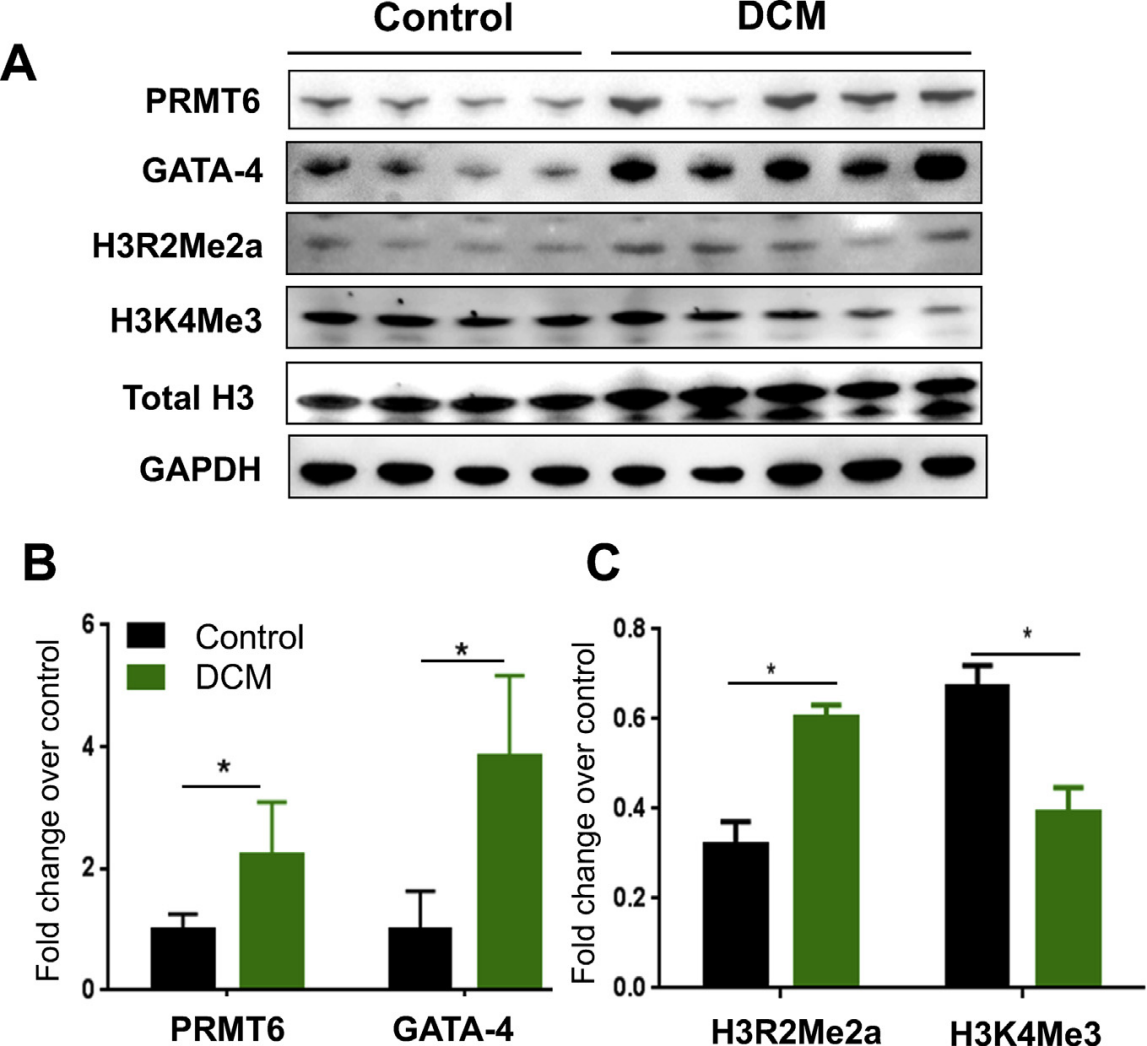
PRMT6 expression is increased in the LV of DCM patients. (A Western blot analysis showing the increase in PRMT6 and H3R2Me2a expression in the heart of DCM patients (n = 5) compared to control hearts (n = 4). GATA-4 expression is shown in parallel. H3K4Me3 is decreased in DCM hearts. Total histone H3 and GAPDH were used as loading control. (B) shows the quantitative analysis of panel (A). (C) H3R2Me2a and H3K4Me3 levels were quantitated to show their reciprocal expression. Statistical significance was calculated by multiple unpaired t-tests assuming fewer assumptions without same scatter (SD) using GraphPad prism and data were graphically represented as mean ± SD. ∗p < 0.05. LV: left ventricle; DCM: dilated cardiomyopathy. The full blot images are available as supplementary material.

In order to identify epigenetic mechanisms implicated in heart failure in human, we assessed several arginine methyltransferases in the LV tissues of control and failing human hearts using Western blot analysis. Results revealed that arginine methyltransferase member 6 (PRMT6) protein expression was significantly higher in the hearts of patients with dilated cardiomyopathy (DCM) (2-fold increase, p = 0.034). The expression of GATA-4, a transcription factor involved in cardiac hypertrophy, was also higher in dilated versus control hearts (Figure 1A & B). Since PRMT6 is known to specifically methylate arginine 2 in histone H3 (H3R2Me2a), we assessed this histone “mark” in LV human hearts. Consistent with the rise of PRMT6 in response to stress, H3R2Me2a increased in failing human hearts compared to control hearts. This increase coincided with a reduction of histone H3 trimethylation at lysine 4 (H3K4Me3) (Figure 1A & C), which is consistent with the mutual exclusion of H3R2Me2a, a “mark” of transcriptional repression, and H3K4Me3 indicative of transcriptional activation.

**Figure 2:**
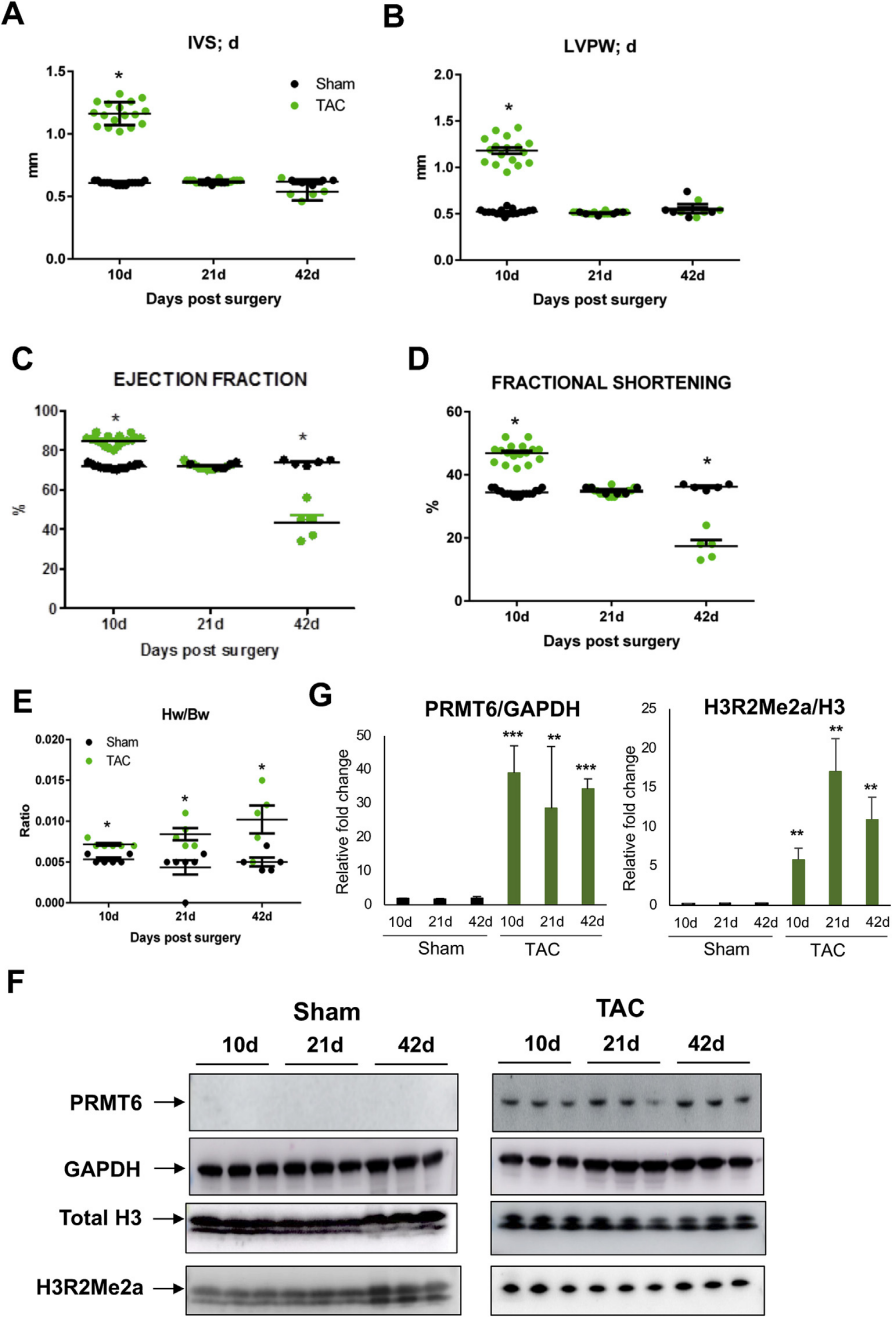
PRMT6 expression is increased in the heart of mice subjected to pressure overload hypertrophy. Echocardiographic measurements were performed in C57BL/6 mice after 10, 21 and 42 days of sham or transverse aortic constriction (TAC) to induce pressure overload hypertrophy. (A) Interventricular septum thickness at diastole (IVS; d), (B) Left ventricular posterior wall thickness at end-diastole (LVPW; d), (C) Ejection fraction and (D) Fractional shortening. (E) At study end point, the animals were euthanized and the hearts were collected. The heart weight/body weight ratio was calculated for each animal. n = 5–17 for control mice, n = 5–17 for TAC mice according to the time-point. Data are represented as means ± SD. Statistical significance was determined using the Holm-Sidak method, alpha = 5.000%. Each time-point was analyzed individually, without assuming a consistent SD. (**F**) Whole heart lysates were prepared and PRMT6 and H3R2Me2a expression were analyzed by Western blot analysis. GAPDH and total histone H3 were used as loading controls. (G) Graph of the quantitative analysis of panel F done with ImageJ software. Data are represented as means ± SD. Statistical significance was determined using Student's t-test. ∗∗p < 0.01, ∗∗∗p < 0.001 were considered significant. The full blot images are available as supplementary material.

The observation that PRMT6 and its epigenetic “mark” H3R2Me2a are highly expressed in the LV of failing human hearts suggests that PRMT6 may play a role in pathological cardiac hypertrophy. Next, we assessed PRMT6 and H3R3Me2a expression in mouse hearts subjected to sham or pressure overload hypertrophy induced by aortic constriction (TAC) for different times. Cardiac dimension and function were recorded by high-resolution echocardiography to monitor the development of hypertrophy in the animals subjected to TAC. As expected, the inter-ventricular septum thickness (IVS; d) and posterior wall thickness (LVPW; d) at diastole significantly increased in mouse hearts after 10 days of TAC surgery compared to sham-operated hearts (Figure 2A & B). At this time-point and consistent with changes normally occurring in the compensatory phase of cardiac hypertrophy, the ejection fraction (EF) and fractional shortening (FS) were significantly higher in mouse TAC hearts compared to sham-operated hearts (Figure 2C & D). At 21 days post-surgery, cardiac dimension and function were normalized in TAC and sham-operated hearts, while hemodynamic parameters significantly declined in TAC mouse hearts at 42 days post-surgery, indicative of cardiac decompensation (Figure 2A-E). Western blot analysis performed from whole heart lysates of three different animals showed a strong increase of PRMT6 at 10 days, 21 days and 42 days post-TAC, which correlated with increased H3R2Me2a (Figure 2F-G). Together, the differential expression of PRMT6 *in vivo* suggests a role of this methyltransferase in cardiac hypertrophy which occurs in the early stage of the cardiac hypertrophy and is sustained afterward.

### 3.2 PRMT6 expression is increased in primary neonatal cardiomyocytes undergoing hypertrophy and PRMT6 over-expression induces hypertrophy

**Figure 3:**
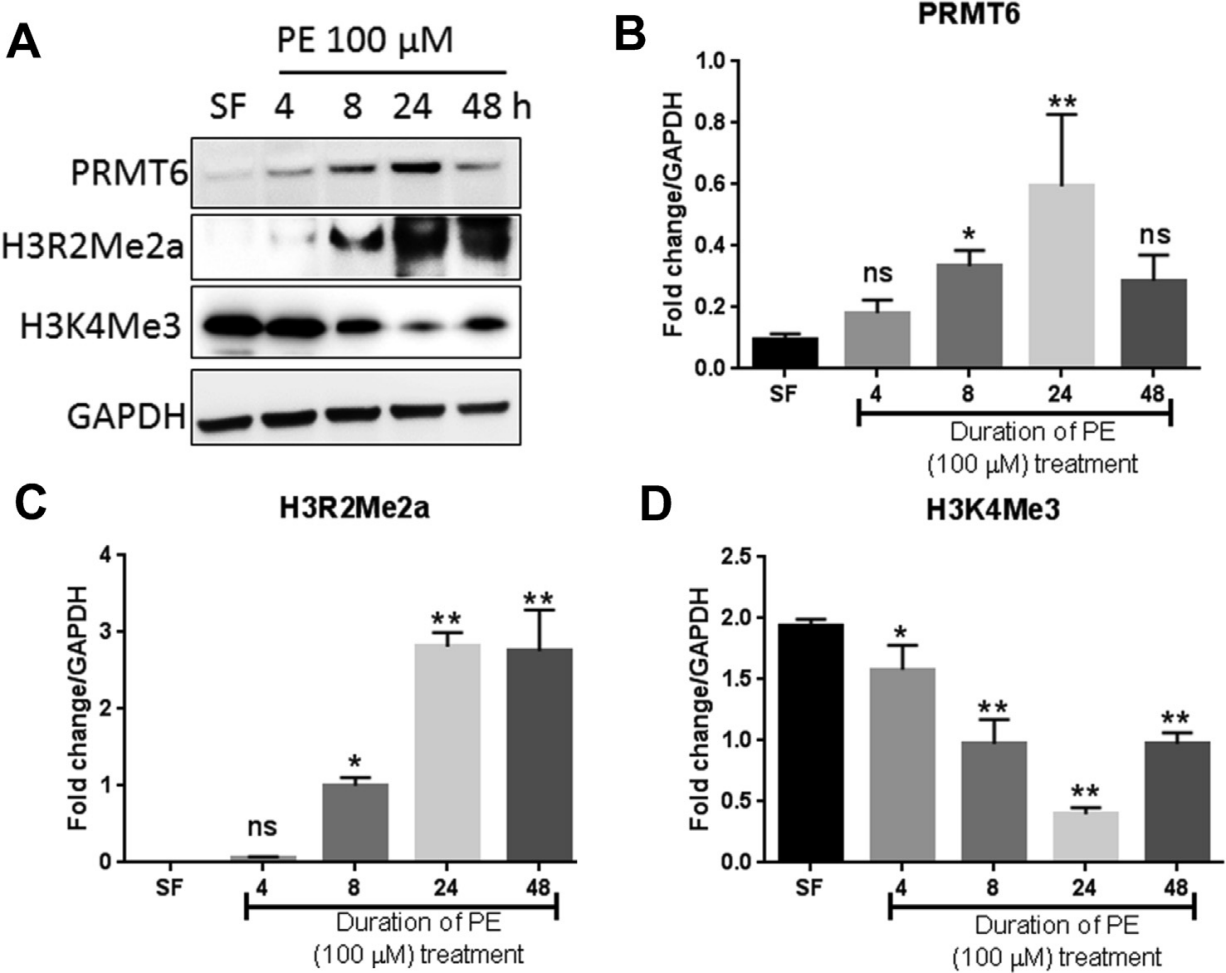
PRMT6 expression is increased in phenylephrine-treated NRVM. Monolayers of rat neonatal cardiomyocytes were treated with phenylephrine (PE, 100μM) for the indicated time periods. (A) Cell lysates were prepared and subjected to Western blot analysis to detect the expression of PRMT6, H3R2Me2a and H3K4Me3. GAPDH is shown as a loading control. (B-D) Quantitative analysis of PRMT6, H3R2Me2a and H3K3Me3 from 3 independent cardiomyocyte preparations. The full blot images are available as supplementary material.

**Figure 4:**
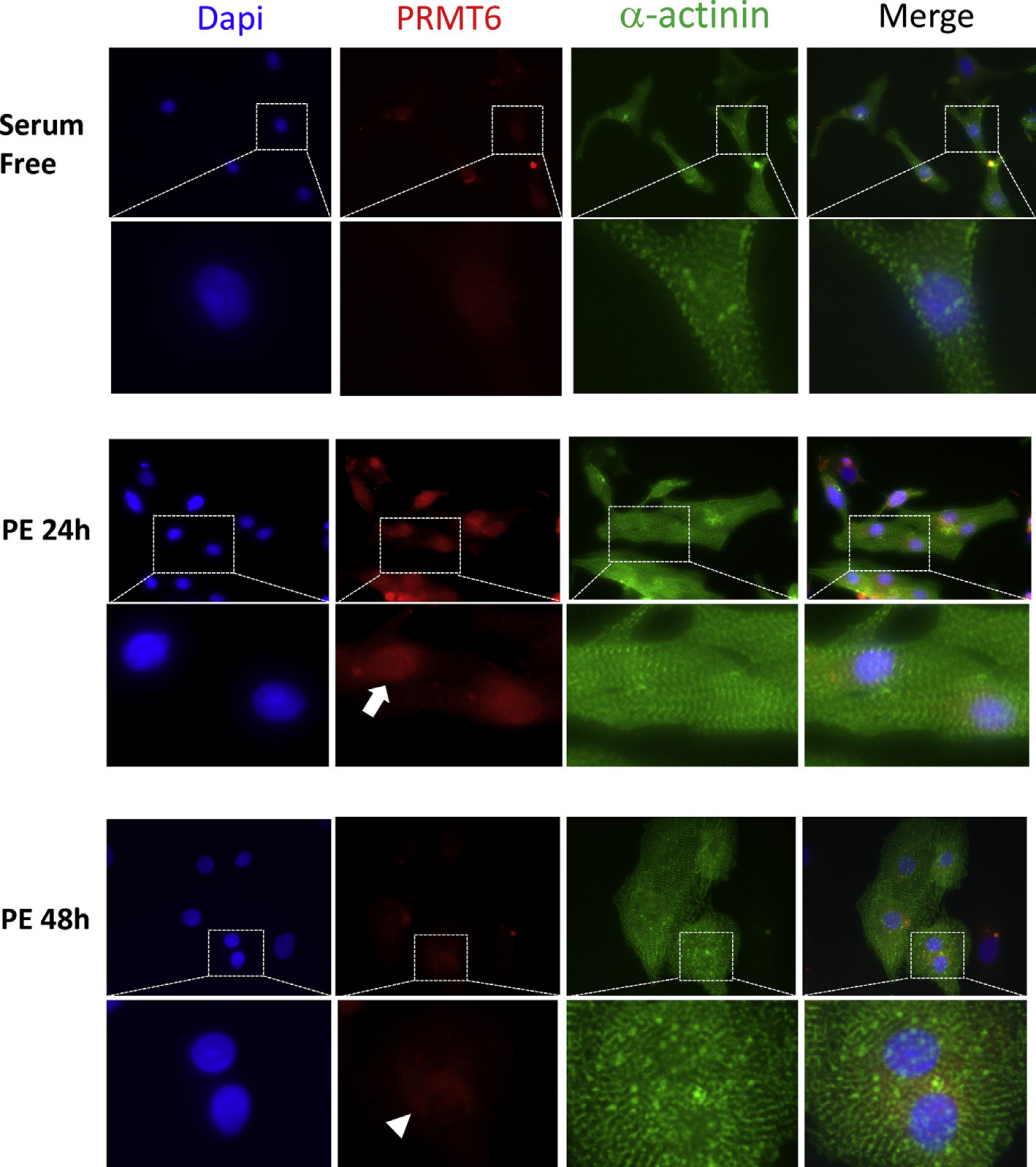
Indirect immunofluorescence showing PRMT6 expression in NRVM maintained in serum-free condition or in media supplemented with PE for 24 and 48 h PRMT6 (red-TRITC), α-actinin (green-FITC), and nuclei counterstained by DAPI are shown as well as the merged images. For each condition, the inlet shows a higher magnification for each image. The arrow shows a prominent PRMT6 localization in NRVM nuclei after 24 h of PE stimulation. The arrowhead shows cytoplasmic PRMT6 in NRVM at 48 h of PE treatment with residual nuclear staining. Images are representative of four independent cardiomyocytes preparations. NRVM: primary neonatal rat ventricular myocytes, PE: phenylephrine.

In order to assess how early PRMT6 dysregulation occurs in response to hypertrophic stimulation, we isolated neonatal rat ventricular myocytes (NRVM) and treated the cells with phenylephrine (PE), a known inducer of cardiac hypertrophy. PE treatment led to a slight increase of PRMT6 as early as 4 h, which further increased and became significant at 8 and 24 h post-PE treatment. After 48 h of PE stimulation, PRMT6 protein expression returned to levels observed after 4 h of PE treatment (Figure 3A & B). H3R2Me2a levels increased incrementally after PE treatment and reached a maximum at 24 h and remained similar at 48 h (Figure 3C). H3K4me3 decreased gradually upon PE treatment up to 24 h and increased thereafter somehow showing an inverse relationship with H3R2Me2a and PRMT6 (Figure 3D). The increase in PRMT6 expression was also observed by indirect immunofluorescence assay. PRMT6 protein was almost undetectable in serum-free condition (Figure 4) and increased progressively after 4 and 8 h of PE treatment (not shown) to reach a maximum at 24 h of PE stimulation. 48 hours post-PE exposure, PRMT6 protein was extremely low (Figure 4). The immunofluorescence assay also revealed that PRMT6 preferentially localized to the nucleus of cardiac cells at 24 h of PE treatment although cytoplasmic expression was also observed. In contrast, PRMT6 localized preferentially to the cytoplasm at 48 h of PE exposure, even though the signal was extremely low at that time of PE treatment (Figure 4).

**Figure 5:**
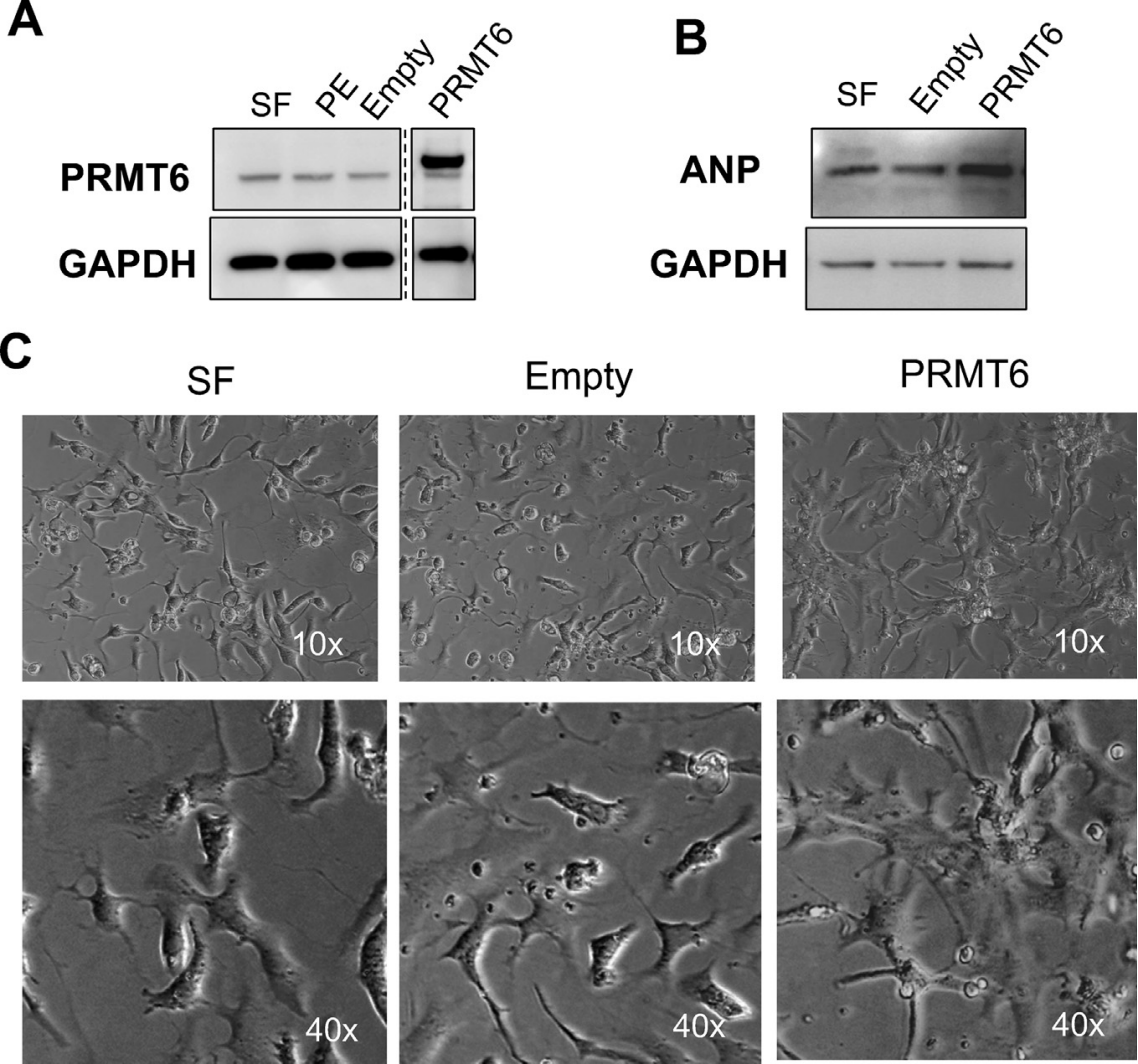
PRMT6 overexpression increases cardiomyocyte hypertrophy. PRMT6 expression vector (PRMT6) or an empty vector (Empty) were transfected into NRVM using Lipofectamine 3000 according to the manufacturer's protocol. After 96 h, total cell lysates were prepared to analyze proteins in Western blot. (A) PRMT6 and (B) ANP protein expressions were measured by Western blot using specific PRMT6 and ANP antibodies. GAPDH was used as loading control. This experiment is a representative of 3 replicates. (C) Phase contrast of NRVM maintained in serum-free condition or transfected with empty vector or PRMT6 expression vector. Images were visualized under phase contrast using a 10x (top panels) or a 40x objective (bottom panels) 96 h after transfection to document cell size. NRVM: primary neonatal rat ventricular myocytes, Empty: empty vector, PRMT6: PRMT6 plasmid, SF: serum-free, PE: phenylephrine at 96 h. The full blot images are available as supplementary material.

Next, in order to study the relationship between PRMT6 and cardiac hypertrophy, we overexpressed this methyltransferase in NRVM using a PRMT6 expression vector and measured the effect on the hypertrophy marker protein, atrial natriuretic peptide (ANP). Overexpression of PRMT6 was successful after the transfection of NRVM (Figure 5A). Forced expression of PRMT6 increased ANP protein expression, suggesting a pro-hypertrophic effect of PRMT6 (Figure 5B). Cardiomyocytes overexpressing PRMT6 also exhibited an increase in size (Figure 5C), as well as an increase in beating rate compared to control cells transfected with an empty vector (data not shown). These data show that PRMT6 can promote cardiac hypertrophy.

### 3.3 Knockdown of PRMT6 inhibits the pro-hypertrophic effect of phenylephrine, which correlates with reduced H3R2Me2a

**Figure 6:**
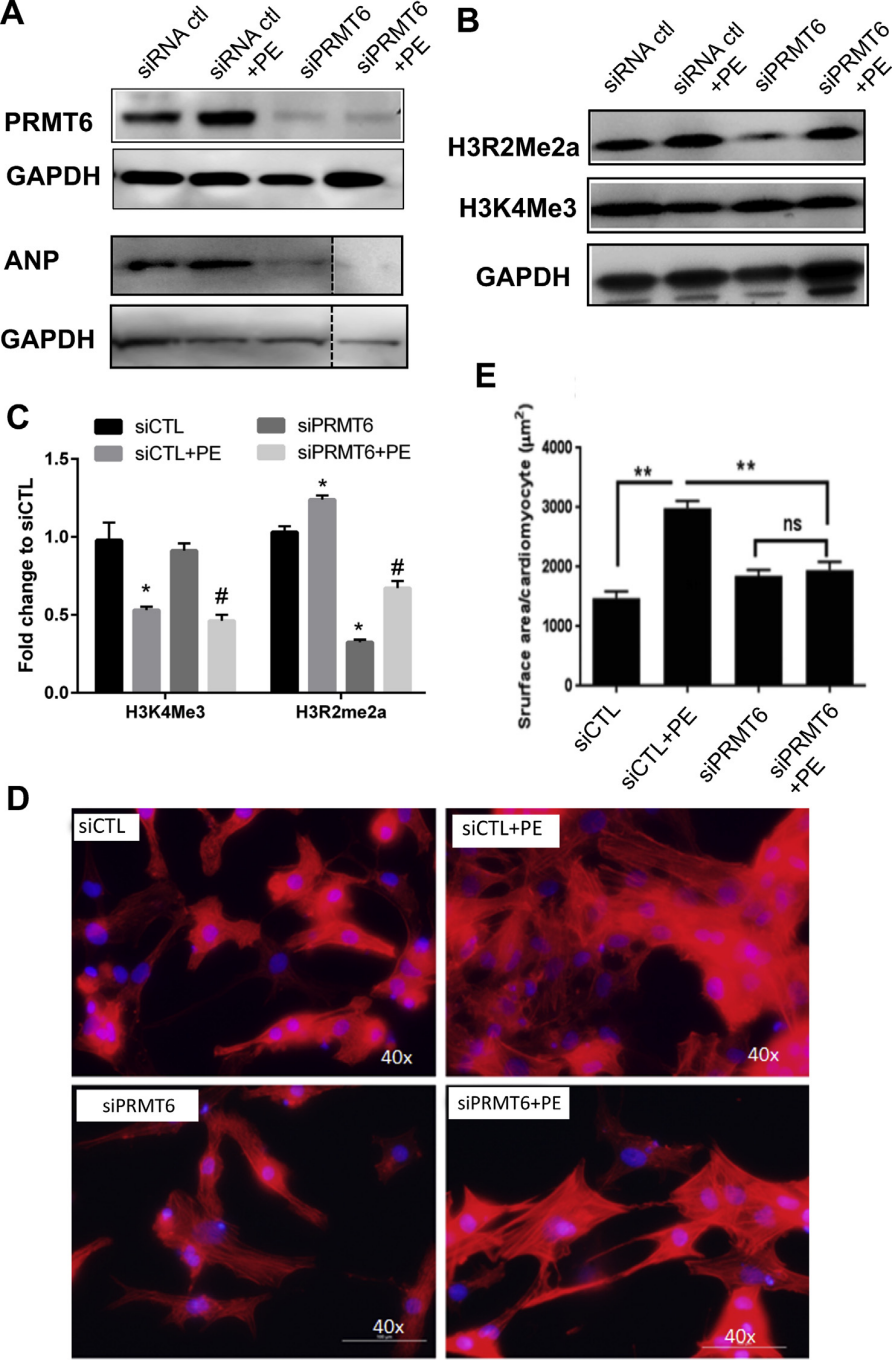
PRMT6 silencing blunts the effect of phenylephrine. NRVM were transfected with PRMT6 siRNA (siPRMT6) or control siRNA (siCTL) as mentioned in Materials and Methods. (A) after 48 h, total cell lysates were prepared and subjected to Western blot analysis to measure PRMT6 and ANP protein expression. GAPDH is shown as loading control. (B) Independent experiments performed to measure histone marks. Cell lysates were analyzed for H3R2Me2a and H3K4Me3 by Western blot. GAPDH was used as loading control. (C) Relationship between H3R2Me2a and H3K4Me3 in cells expressing normal or low PRMT6 maintained in serum-free condition or treated with PE. (D) NRVM were cultured in 6-well plates and transfected with control siRNA or PRMT6 siRNA. After transfection, cells were maintained in serum-free condition or challenged with 100 μM PE. After 24 h, the cells were stained with TRITC-labelled phalloidin for F-actin. Cells were visualized by fluorescence microscopy. (**E**) Cell surface area was measured in at least 5 different fields using a 40x objective using Adobe acrobat CS6. Data are expressed as mean ± SD. Statistical analysis was performed by One-way ANOVA (panel E) or two-way ANOVA (panel C) followed by Tukey's multiple comparison test, with ∗p < 0.05. The full blot images are available as supplementary material.

To establish the role of PRMT6 in cardiomyocyte hypertrophy, we used RNA interference to silence PRMT6 expression in NRVM. As shown in Figure 6A, the transfection of NRVM with PRMT6 siRNA significantly reduced PRMT6 protein expression. Consistent with the effect of PRMT6 in H3R2Me2a, knockdown of PRMT6 significantly reduced H3R2Me2a. In serum-free condition, silencing of PRMT6 resulted in a 75% decrease of H3R2Me2a expression, while in PE-treated cells, PRMT6 silencing lead to a 50% reduction in H3R2Me2a compared to cells transfected with siControl (Figure 6B & C). No significant change was observed for H3K4Me3 in cells expressing normal or reduced PRMT6 expression suggesting that PRMT6 is required for H3R2Me2a but not for H3K4Me3 (Figure 6B & C). Silencing of PRMT6 in serum-free condition had no significant effect on cardiomyocyte size. Conversely, knock-down of PRMT6 in PE-cells significantly reduced cell size by almost 50% compared to cells expressing normal level of PRMT6 (Figure 6D & E). Altogether, these results indicate that PRMT6 is required for PE-mediated cellular hypertrophy and this effect involves H3R2Me2a.

### 3.4 PRMT6 silencing antagonizes the effect of PE on beating rates and electrophysiological properties of cardiomyocytes

**Figure 7:**
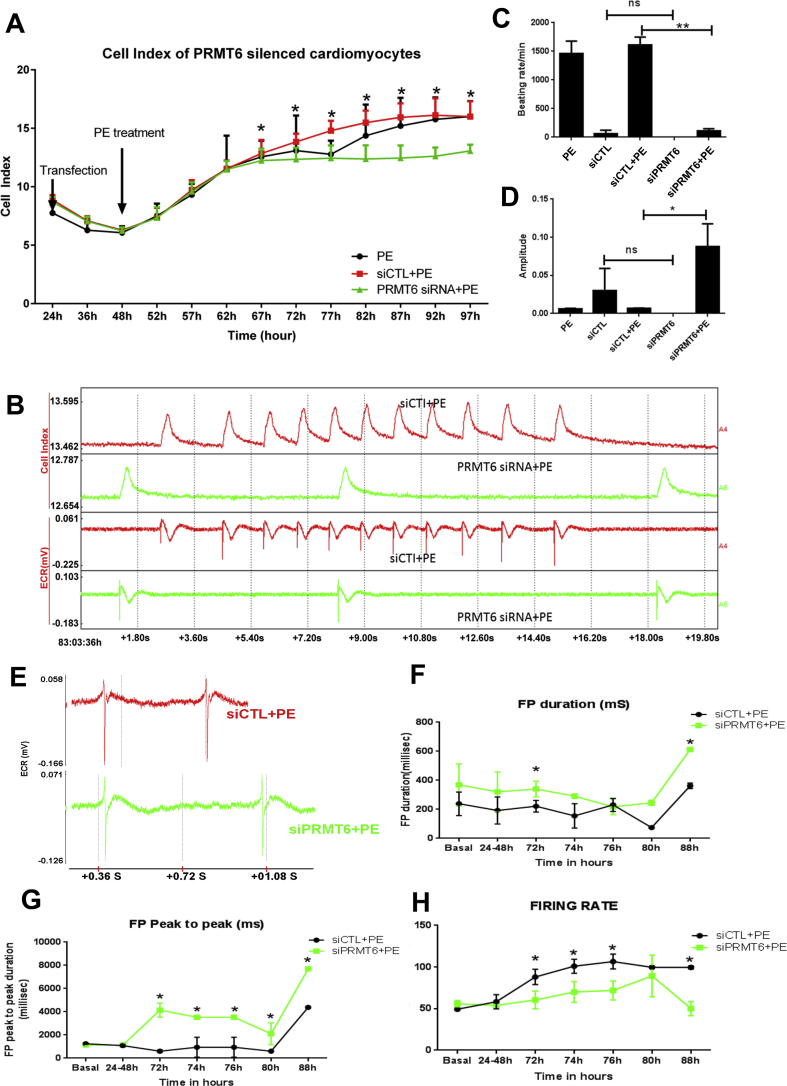
PRMT6 silencing blunts the effect of phenylephrine on contraction rates. Cardiomyocytes were plated in ECR Cardioplates (48 wells) at a density of 30,000 cells/well. After 24 h, cells were transfected with PRMT6 siRNA (siPRMT6) or control siRNA (siCtl) and 24 h later, PE (100μM) treatment was started and maintained for the indicated time. (A) The cell index was monitored in real-time for 97 h using the RTCA CardioECR system that measures contraction and global ion channel activities. (B and C) Quantitative analysis of beating rates and amplitude of contraction of NRVM at 83 h. PE: untransfected cells treated with PE, siCTL: serum-free treated cells transfected with siControl, siCTL + PE: cells transfected with siControl and treated with PE, siPRMT6: serum-free treated cells transfected with siPRMT6, siPRMT6+PE: cells transfected with siPRMT6 and treated with PE. (D) Snapshot of real-time contractility and ion channel function at 83 h. (**E**) Extracellular field potential traces in NRVM transfected with siControl or siPRMT6 and treated with 100 μM PE. (**F**) Measurement of the field potential duration of NRVM transfected with siControl or siPRMT6 after PE treatment. **(G)** Extracellular field potential “peak to peak” intervals measured in NRVM transfected with siControl or siPRMT6 and treated with PE for the indicated times showing significantly longer intervals in siPRMT6 cells compare to siControl cells. **(H)** Firing rates measurements for the indicated time showing significantly reduced firing rates of siPRMT6 cells compared to siControl cells. Data are represented as mean ± SD, and the statistical analysis was performed by two-way ANOVA (panel A) or one-way ANOVA (panel B, C, F–H) followed by Tukey's multiple comparison test, with ∗p < 0.05.

Next, we assessed the effect of PRMT6 on cardiac cell contraction and relaxation cycles. For this, we used the RTCA-cardio system (ACEA Biosystems), a microelectronic sensor-based system that monitors cell contractility and global electrical activity in live cells. Microelectrodes measure short-term (msec to sec) and long-term (days to weeks) impedances during contraction-relaxation cycles, providing accurate measurements of cell viability, contractility and global ion channel activity of cardiomyocytes in real-time [31]. As expected, PE treatment increased cardiomyocyte size as indicated by an increase in the cell index compared to cells maintained in serum-free condition. From 68h to 98h after cell plating (43–73 h post-transfection), the cell index was significantly reduced in PRMT6 silenced cells (siPRMT6) compared to cells transfected with control siRNA (siControl) (Figure 7A). This result shows that PRMT6 is critical for the pro-hypertrophic effect of PE on NRVM. Short-term recording over an interval period of 20 s at 83 h after plating the cells, showed that the silencing of PRMT6 dramatically reduced the beating rates of NRVM until the end of the recording (Figure 7B & C). Amplitudes of contractions were significantly higher in PE-treated cells transfected with PRMT6 siRNA compared to siControl transfected cells (Figure 7B & D). Close recording of global field potentials during a shorter time interval, showed a significant increase by almost 2-fold of the field potential duration in PRMT6 silenced cells compared to siControl at 72h and 88h (Figure 7E & F). In addition, consistent with the increased beating rate of siPRMT6 cells, the “peak to “peak” intervals of the field potentials were significantly longer in siPRMT6 cells compare to siControl cells (Figure 7E & G). Measurement of the firing rate of the cells, which records the number of extracellular field potentials per minute, showed significantly reduced firing rates of siPRMT6 cells compared to siControl cells (Figure 7H), indicative of a reduced global ion channel activity. Altogether, these data show that reducing PRMT6 expression impairs the contraction rate and the global field potentials of NRVM stimulated with PE. Thus, we conclude that PRMT6 is critical for the maintenance of cardiac contractility and ion channel activity of NRVM.

## 4 Discussion

**Figure 8:**
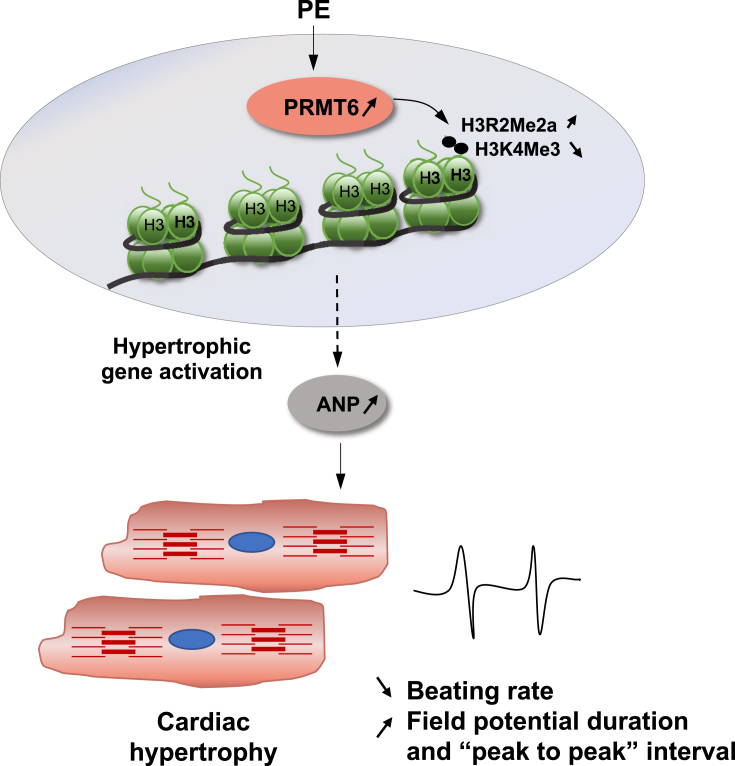
Model depicting the role of PRMT6 in PE-induced cardiac hypertrophy. In response to PE stimulation, PRMT6 is upregulated in cardiac cells, which increases its signature histone methylation “mark” H3R2Me2a and reduces H3K4Me3. These histone posttranslational modifications may activate hypertrophic genes such as ANP directly or indirectly, resulting in cardiac hypertrophy. Over time, these changes have profound effects on cardiac beating rates and global electrical activity, indicating that PRMT6 is required for the PE-mediated hypertrophic effect and to maintain contractility as well as global electrical activity of cardiac cells.

The main finding of our study is that PRMT6 is critical for the hypertrophic response mediated by PE in NRVM. Assessment of PRMT6 in human heart specimen show higher level of the methyltransferase in dilated human cardiomyopathic hearts compared to control hearts, which is recapitulated in mouse hearts after pressure overload hypertrophy, and in NRVM stimulated with PE for 8 and 24 h PRMT6 up-regulation in response to cardiac stress *in vivo* or after agonist treatment is associated with an increase in its signature histone methylation mark, H3R2Me2a. Specific silencing of PRMT6 blunts the increased cardiomyocyte size mediated by PE, while transient over-expression of PRMT6 has the opposite effect. Reducing PRMT6 expression has a profound effect on PE-induced cardiac beating rates and global electrical activity. These results indicate that adequate PRMT6 expression is important for cardiac cell homeostasis and function (Figure 8).

PRMT members have been investigated in several diseases especially cancer [32, 33, 34]. With the exception of one report documenting the protective role of PRMT1 against heart failure through inactivation of calcium/calmodulin-dependent protein kinase II [27], the role of arginine methyltransferases in the pathogenesis of cardiac hypertrophy remains unexplored. In order to understand mechanisms implicated in pathological cardiac remodeling, we assessed the expression of PRMTs in failing human hearts and control hearts. Among the PRMT members, PRMT6 protein expression was significantly increased in dilated human hearts compared to control hearts. Rather than a consequence of the cardiomyopathy, PRMT6 up-regulation is likely to contribute to the development of the disease. Indeed, the assessment of PRMT6 in a pressure overload model in mice showed a strong up-regulation of PRMT6 protein during the adaptive phase of cardiac hypertrophy after 10 days of aortic constriction. PRMT6 protein level remained high after 21 and 42 days of TAC. In NRVM, PRMT6 protein expression increased as early as 4 h after agonist treatment, further increased at 8 h of PE treatment, which preceded cellular hypertrophy. Subsequently, PRMT6 expression continued to rise to reach a maximum after 24 h of PE challenge and then returned to basal levels at 48 h. These results indicate an early response of PRMT6 to hypertrophic stimulation and that compensatory mechanisms likely occur after 48 h of PE stimulation. Forced expression of PRMT6 enhanced the hypertrophic response as shown by increased expression of the hypertrophic marker ANP and cellular enlargement. Importantly, reduced expression of PRMT6 inhibited the effect of PE on cell size. Altogether, these results indicate a critical role of PRMT6 in cardiomyocyte hypertrophy.

Our data indicate that the pro-hypertrophic effect of PRMT6 is mediated, at least in part, by H3R2Me2a. PRMT6 is the primary enzyme responsible for the arginine methylation of H3 [28,29]. As a type II PRMT enzyme, PRMT6 mediates the asymmetric methylation of H3 on arginine 2. Accordingly, forced expression of PRMT6 in NRVM enhanced H3R2Me2a, a mark normally associated with transcriptional repression. Reducing PRMT6 levels inhibited H3R2me2a, as well as cellular hypertrophy, indicating that the PE-mediated effect of PRMT6 implicates H3R2Me2a. H3R2Me2a is known to antagonize H3K4Me3 [28,29]. Since our data show an inverse relationship between the 2 histone marks H3R3Me2a and H3K4Me3 up to 24 h of PE stimulation, it is reasonable to speculate that PRMT6 drives hypertrophy by increasing H3R2Me2a deposition, which in turn inhibits genes that are normally repressed. One known PRMT6/H3R2Me2a repression targets is thrombospondin-1 (TSP-1) [35]. Thus, its role in PRMT6 mediated cardiac hypertrophy should be investigated in the future. Our results also showed that after 48 h of PE treatment, H3R2Me2a remained high while PRMT6 expression was reduced. H3K4Me3 levels decreased overtime up to 24h of PE treatment and increased at 48 h. These observations suggest that compensatory mechanisms may be activated in the late phase of cardiac hypertrophy (48h) in order to block the hypertrophic effect of PE. Alternatively, other PRMTs or histone modifying enzymes may be activated at 48h of PE stimulation to maintain H3R2Me2a and H3K4Me3 levels. In fact, we know that the expression of one other PRMT remains high after 48h of PE stimulation (data not shown). PRMT6 is also responsible for H4R3me2a and H2AR3me2a methylation, which are associated with actively transcribe gene promoters [30]. Because it is well established that the histone “marks” act in concert, it is likely that PRMT6 catalyzes additional histone “marks” such as H4R3me2a and H2AR3me2a, which in turn may activate hypertrophic responsive genes. Therefore, it will be important to identify the specific gene programs regulated by PRMT6 and other PRMTs as well as the specific histone “marks” regulated by PRMT6 in the heart in subsequent studies.

The vast majority of studies document PRMT6 expression predominantly in the cell nucleus. PRMT6 translocates to the nuclear compartment in a gain-of-function study in mice where it acts as a coactivator of NF-kB [36]. PRMT6 can also form a complex with transcription factors like RUNX1 [37], which prompted us to assess RUNX1 expression in cells stimulated with PE. Western blot and immunofluorescent analyses revealed no significant change in RUNX1 protein expression (data not shown). In hematopoietic progenitor cells, PRMT6 is part of a repressor complex with RUNX1 and Sin3a, replacing HDAC1 and PRMT1, and releasing the MLL/WDR5 complex. This in turn, reduces the H3K4Me3 mark with a concomitant increase in H3R2Me2a [37]. On this basis, we measured RUNX1 and Sin3a in the LV of control and failing human hearts but found no significant differences between the two groups of hearts (data not shown). Global expression of the two factors may not differ in failing hearts but their recruitment at specific genomic regions and protein complex formation may vary. Thus, interaction between RUNX1, Sin3a and PRMT6 should be investigated further as well as binding of the complex to chromatin in response to hypertrophic stimulation. Also, our immunofluorescence data showed PRMT6 in nuclear regions with a prominent nuclear staining and even cytoplasmic presence after hypertrophic stimulation, indicating that the subcellular localization of PRMT6 is not strictly nuclear. Consistent with this, PRMT6 can be detected in the cytoplasm of cancer cells. For instance, cytoplasmic substrates of PRMT6 include CREB Regulated Transcription Coactivator 2 (CRCT2) sequestrated in the cytoplasm under basal condition, G Protein Pathway Suppressor 2 (GPS2) and CRAF, which can localize to the cytoplasm and also to the cell membrane [38]. The observation that PRMT6 localizes to regions other than the cell nucleus is interesting. It suggests that PRMT6 could methylate proteins other than histones or chromatin-associated proteins, which maybe a topic of future investigation.

Non-invasive recording of cell impedances allows the measurement of beating rates and of global field potentials, which are good indicators of action potentials measured by electrophysiological patch-clamp [39]. Therefore, we analyzed the beating rates, the duration of the field potential as well as the “peak to peak” interval of NRVM expressing normal or low-level PRMT6. Our data show that cardiomyocytes expressing reduced levels of PRMT6 exhibit slower beating rates and prolongated global field potentials, indicating that PRMT6 is critical for the beating and ion channel activity of NRVM. Changes in global field potentials indicate an increased depolarization possibly due to prolonged activation of I_Na_ (late) or I_Ca_ channels. Alternatively, reduced repolarization due to reduced K^+^ efflux could explain the prolongation of the field potential in cells with low PRMT6. In line with this, the stability of the H3K4Me “mark” is crucial for the regulation of the potassium voltage-gated channel interacting protein 2 (Kcnip2). Lack of Kcnip2 in cardiomyocytes causes prolongation of action potentials [40]. Therefore, individual ion channels should be investigated using the patch-clamp technique to assess the role of PRMT6 on electrical properties with high accuracy.

In summary, we show a critical role of PRMT6 in maintaining cardiomyocyte contractile properties, associated with H3R2Me2a. These results underline the importance of keeping low-level PRMT6 to prevent cardiomyocyte hypertrophy. Whether similar mechanisms are observed *in vivo* needs to be investigated. This study may provide the rationale to design novel therapeutic targets to treat heart failure in human.

## Declarations

### Author contribution statement

V. Raveendran: Conceived and designed the experiments; Performed the experiments; Analyzed and interpreted the data; Wrote the paper.

C. Poizat: Conceived and designed the experiments; Analyzed and interpreted the data; Wrote the paper.

Q. Al Haffar and M. Kunhi: Performed the experiments; Analyzed and interpreted the data.

K. Behalj: Performed the experiments.

W. Al-Habeeb, J. Al-Buraiki and A. Eyjolsson: Contributed reagents, materials, analysis tools or data.

### Funding statement

This work was supported by the 10.13039/501100002382King Faisal Specialist Hospital & Research Centre institutional funds. C. Poizat was supported by the 10.13039/501100004919King Abdulaziz City for Science & Technology (grant 13-MED456-20) and the Masonic Medical Research Institute.

### Competing interest statement

The authors declare no conflict of interest.

### Additional information

No additional information is available for this paper.
