# Supplementary material for: Protein arginine methyltransferase 6 mediates cardiac hypertrophy by differential regulation of histone H3 arginine methylation
Source: Heliyon. 2020 May 12;6(5):e03864. doi: 10.1016/j.heliyon.2020.e03864 (PMC7218648; doi:10.1016/j.heliyon.2020.e03864)
Supplement: Supplementary file 1 — Supplementary Material [file mmc1.pdf]

Supplementary files

Original blots for Figure 1

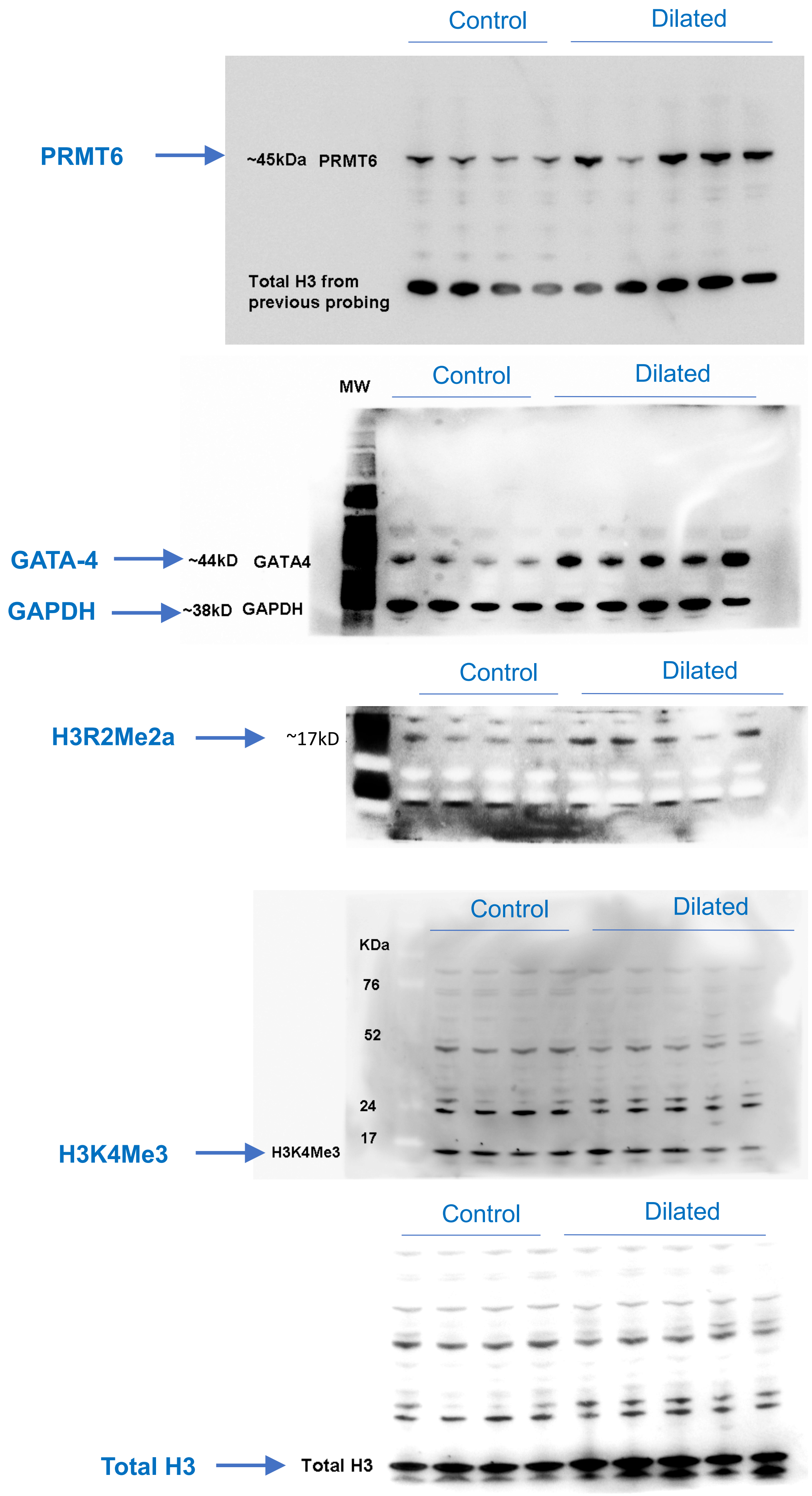

Original blots for Figure 2

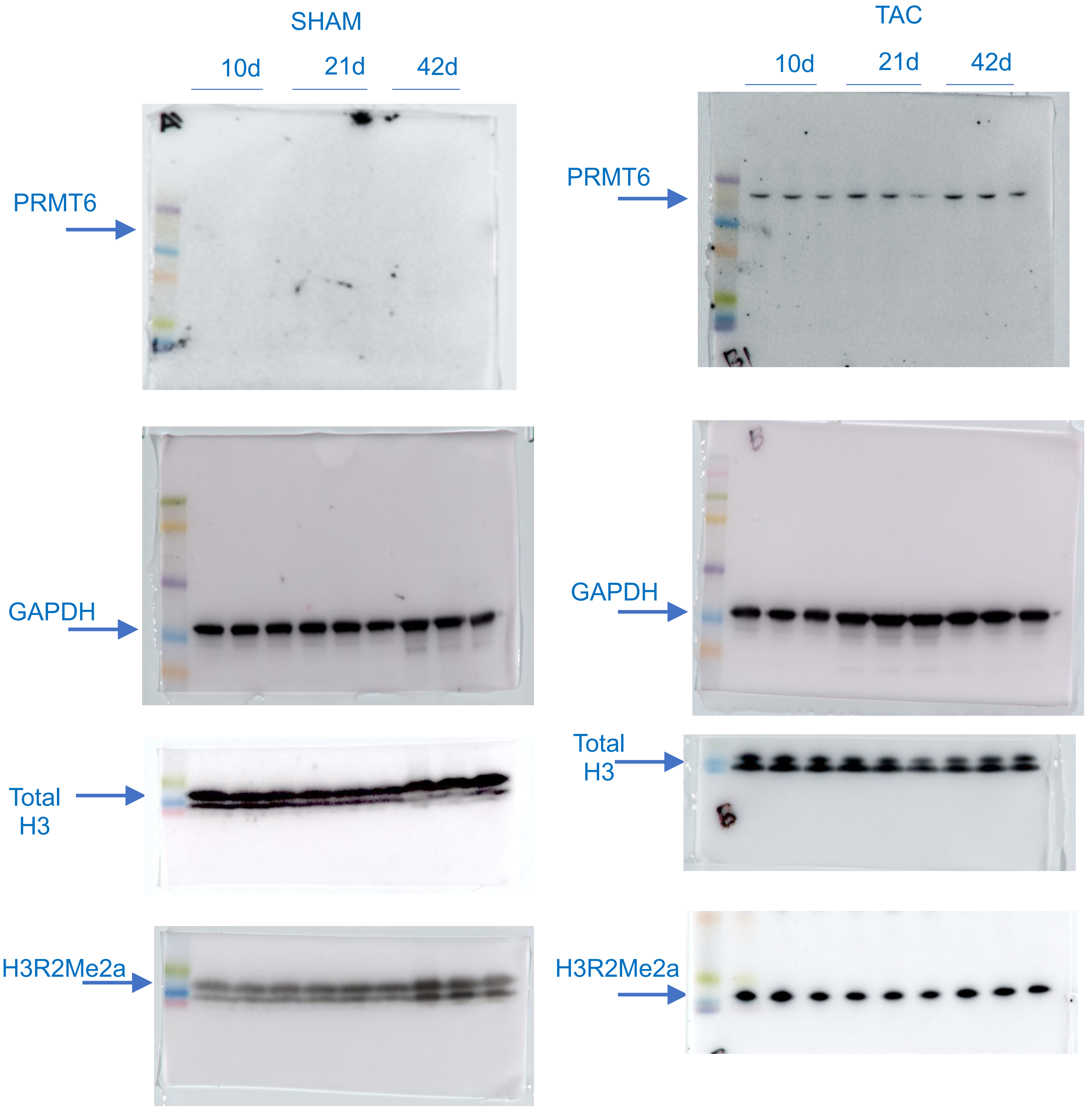

Original blots for Figure 3

1 2 3 4 5

PRMT6

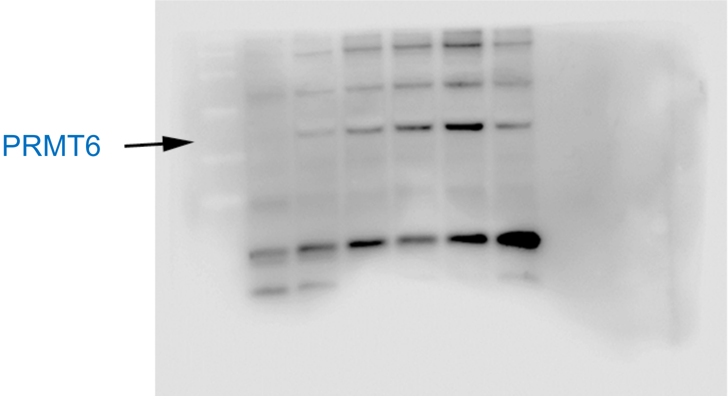

1 Serum free
2 PE 4h
3 PE 8h
4 PE 24h
5 PE 48h

H3R2Me2a

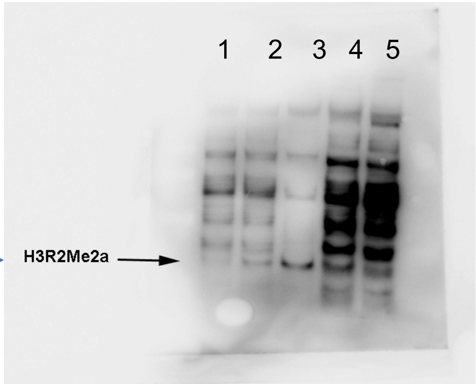

H3K4Me3

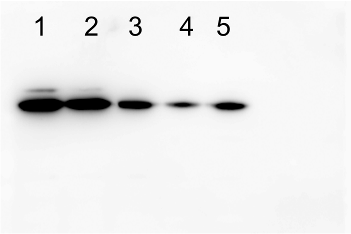

GAPDH

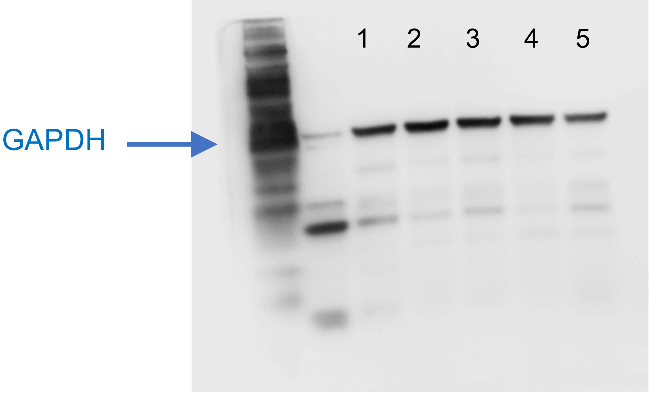

Original blots for Figure 5

Panel A

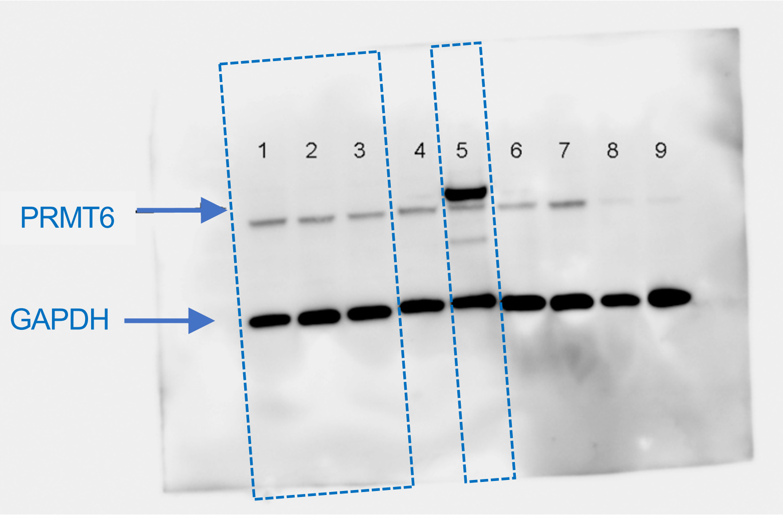

1. Serum free
2. PE
3. Empty vector
4. PRMT8 vector
5. PRMT6 vector
6. siControl
7. siControl + PE
8. siPRMT6
9. siPRMT6+PE

Panel B

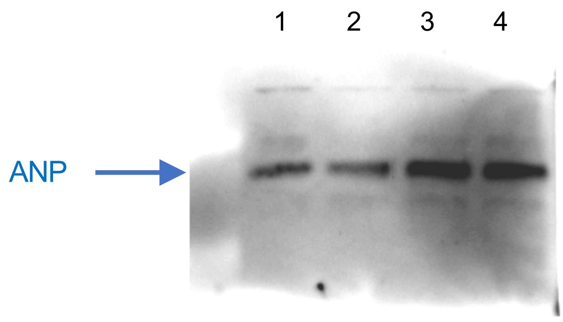

1 Serum free
2 Empty vector
3 PRMT6
4 PRMT8

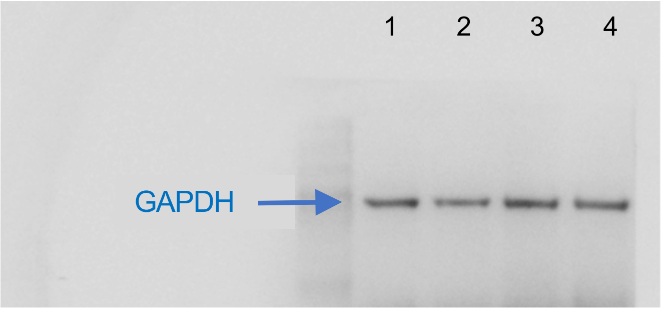

Original blots for Figure 6

Panel A

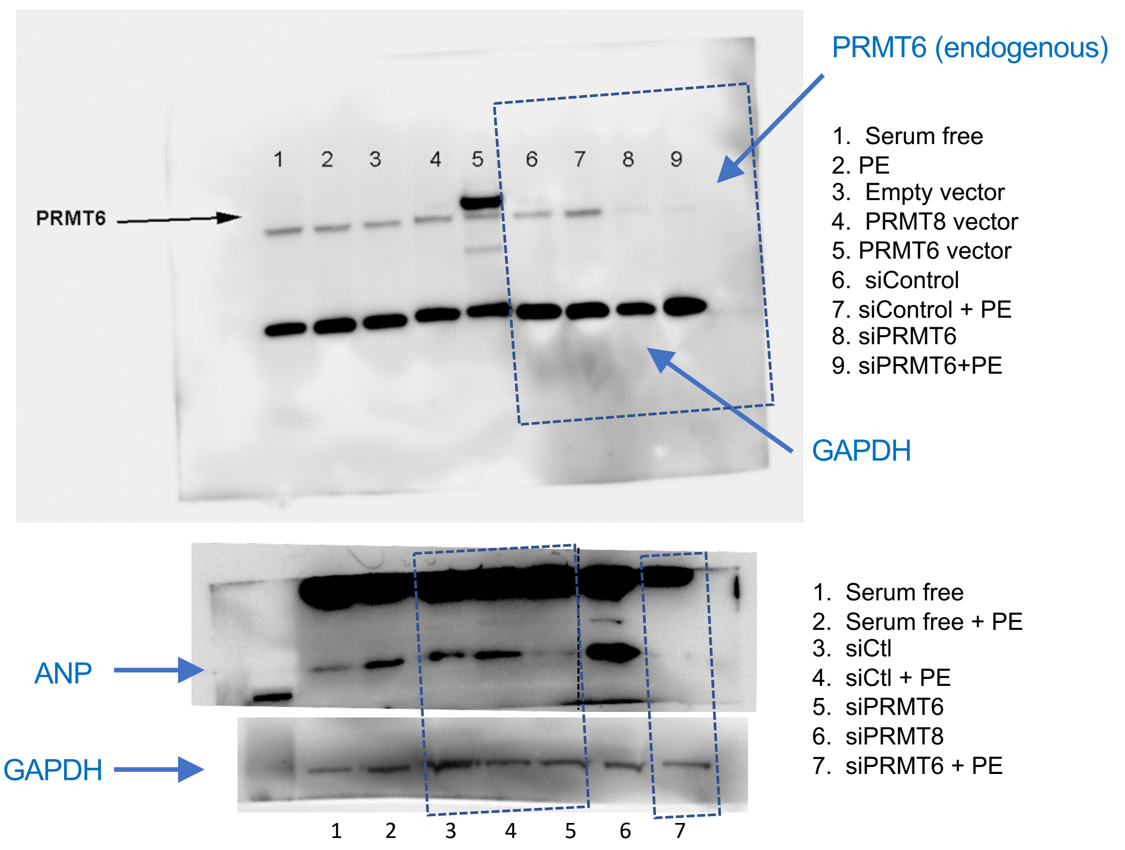

Panel B

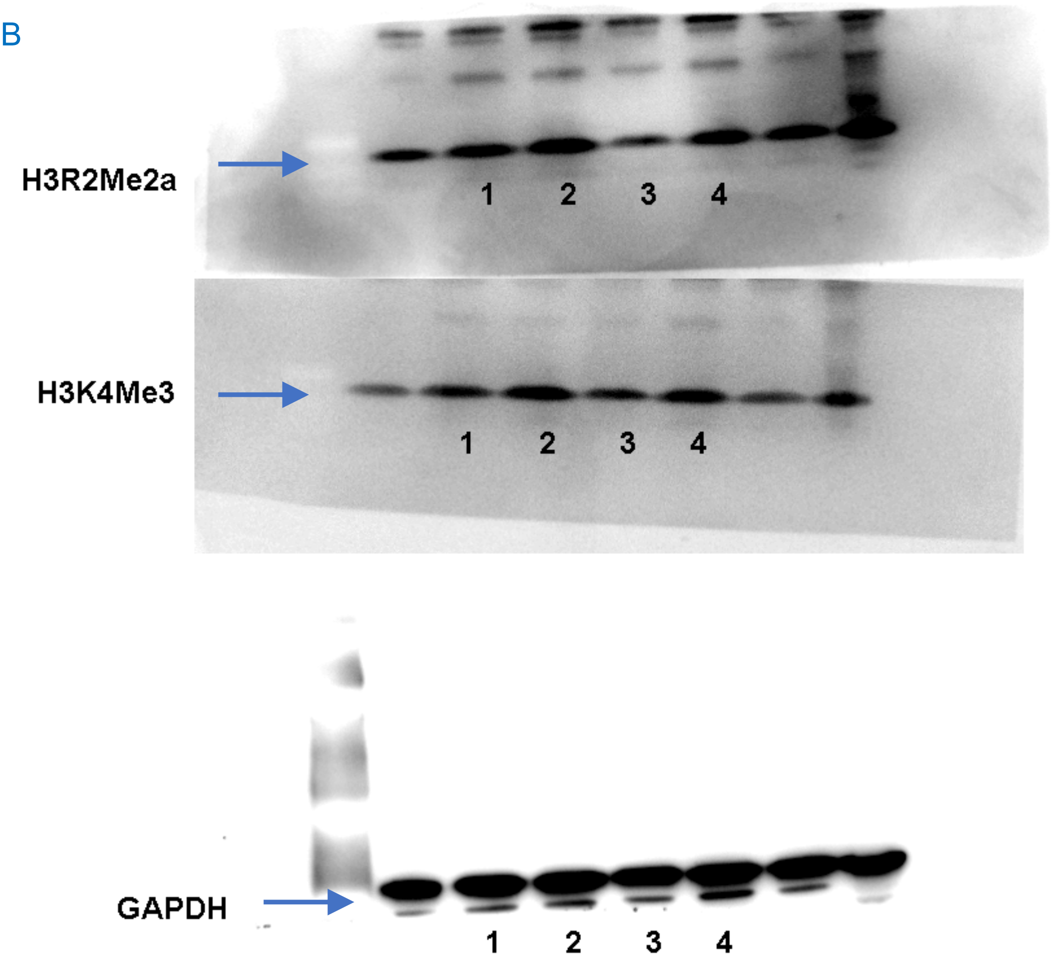

1 siCTL; 2 siCTL+PE; 3 siPRMT6; 4 siPRMT6+PE
